# Modeling of age-dependent amyloid accumulation and γ-secretase inhibition of soluble and insoluble Aβ in a transgenic mouse model of amyloid deposition

**DOI:** 10.1002/prp2.12

**Published:** 2013-12-05

**Authors:** Joanna Parkinson, Bart Ploeger, Paulina Appelkvist, Anna Bogstedt, Karin Dillner Bergstedt, Susanna Eketjäll, Sandra A G Visser

**Affiliations:** Primary laboratory of origin, AstraZeneca R&D CNSP Innovative MedicinesSE-15185, Södertälje, Sweden

**Keywords:** Alzheimer's disease, Aβ, disease progression, modeling, pharmacokinetic-pharmacodynamic, Tg2576 mouse, γ-secretase inhibitor

## Abstract

According to the “amyloid hypothesis,” accumulation of amyloid beta (Aβ) peptides in the brain is linked to the development of Alzheimer's disease. The aims of this investigation were to develop a model for the age-dependent amyloid accumulation and to quantify the age- and treatment-duration-dependent efficacy of the γ-secretase inhibitor MRK-560 in the Tg2576 transgenic mouse model of amyloid deposition. Soluble and insoluble Aβ40 and Aβ42 brain concentrations were compiled from multiple naïve, vehicle, and MRK-560-treated animals. The age of Tg2576 mice in the studies ranged between 3.5 and 26 months. Single doses of MRK-560 inhibited soluble Aβ40 levels in animals up to 9 months old. In contrast, MRK-560 did not cause significant acute effects on soluble Aβ40 levels in animals older than 13 months. Absolute levels of Aβ variants increased exponentially over age and reached a plateau at ∼20 months. In the final model, it was assumed that MRK-560 inhibited the Aβ production rate with an Aβ level-dependent IC_50_.The age-dependent increase in Aβ levels was best described by a logistic model that stimulated the production rate of soluble Aβ. The increase in insoluble Aβ was defined as a function of soluble Aβ by using a scaling factor and a different turnover rate. The turnover half-life for insoluble Aβ was estimated at 30 days, explaining that at least a 4-week treatment in young animals was required to demonstrate a reduction in insoluble Aβ. Taken together, the derived knowledge could be exploited for an improved design of new experiments in Tg2576 mice.

## Introduction

Alzheimer's disease (AD) is the most common form of dementia affecting memory, behavior, and cognition. It is a progressive disease that eventually leads to death (Alzheimer's Association, 2012). Currently, over 35 million people worldwide have AD (Querfurth and LaFerla 2010). At present, there is no effective treatment available and there exists a large unmet medical need for new, effective, and safe medicines (D'Onofrio et al. 2012). Although the precise mechanism of AD is unknown, evidence supports the amyloid hypothesis which implicates a link between the pathogenesis of the disease, elevated levels of amyloid beta (Aβ) peptide and its aggregation in the brain (Hardy and Selkoe 2002; Hardy 2009; Karran et al. 2011; Walsh and Teplow 2012).

Amyloid beta peptides, including Aβ40 and Aβ42 variants, are produced by a sequential cleavage of the amyloid precursor protein (APP) by β- and γ-secretases (Lichtenthaler et al. 2011). According to the amyloid hypothesis, reduction in Aβ production may result in slowing or stopping the development of AD. Consequently, inhibitors or modulators of β- and γ-secretases became prime targets for disease-modifying treatments in AD (Citron 2010; Golde et al. 2010; Panza et al. 2011; D'Onofrio et al. 2012; Wolfe 2012). To date, all tested γ-secretase inhibitors (GSI) failed in clinical trials (Kreft et al. 2009; Imbimbo and Giardina 2011; Wolfe 2012). A recent model-based meta-analysis showed that the cerebrospinal fluid (CSF) Aβ inhibition was not sufficient at the used doses in these trials (Niva et al. 2013). Moreover, preclinical studies could provide an opportunity to test hypotheses about targetable disease processes and to derive valuable information on the efficacy of disease-modifying treatments. Preclinical experiments may support the selection of candidate compounds, the prediction of human dose, and the design of early clinical studies (Niva et al. 2013). A prerequisite is the application of a quantitative analysis (such as pharmacokinetic-pharmacodynamic [PKPD] modeling) in order to understand the dynamics and the interspecies translation of Aβ as biomarker for secretase inhibition within the brain, plasma, and CSF compartments.

Multiple transgenic mice models were developed following the discovery of the apparent role of APP in AD. One transgenic mice model, Tg2576, overexpresses the Swedish mutation variant of human APP (Hsiao et al. 1996). This mutation has been linked to the greater proteolytic cleavage of APP and to an altered cellular mechanism of Aβ generation (Haass et al. 1995). Consequently, Tg2576 mice produce highly elevated Aβ levels followed by the formation of Aβ plaques, as well as age-dependent behavioral, learning, and memory impairment (Hsiao et al. 1996; Chapman et al. 1999; Kawarabayashi et al. 2001; Sasaki et al. 2002; Middei et al. 2006). The Tg2576 mouse, an established preclinical model of AD, has its limitations. It is primarily a model of Aβ accumulation and its subsequent deposition which are not the sole factors in the Alzheimer's pathology. Other hallmarks of AD, such as tau pathology, inflammation, neuronal cell death, and brain atrophy are not present in the Tg2576 model (Brunden et al. 2009; Cameron and Landreth 2010). It is, therefore, important to note that changes in Aβ levels may only reflect a part of the AD progression.

The first objective of the present investigation was to develop a mathematical model for the age-dependent amyloid accumulation in soluble and insoluble Aβ observed in the Tg2576 mice. Although age-dependent changes in Aβ levels have been described qualitatively, to our knowledge no mathematical model exists. The second objective was to quantify the age- and treatment-duration-dependent efficacy of the GSIN-[cis-4-[(4-chlorophenyl)-sulfonyl]-4-(2,5-difluorophenyl)cyclohexyl]-1,1,1-trifluoromethanesulfonamide (MRK-560) (Churcher et al. 2006). To achieve these goals, we have developed a PKPD model that incorporates (1) the MRK-560 pharmacokinetics, (2) the PKPD relationship, (3) the Aβ turnover, (4) and the amyloid progression (AP). The model was able to describe the decrease in efficacy of GSI over age, the lack of effect in old animals (Das et al. 2012), and that chronic treatment is needed to reduce insoluble brain Aβ levels (Wang et al. 2012).The quantitative understanding of Aβ dynamics affected by age and treatment duration, could help in understanding the opportunities and limitations of the Tg2576 mouse model in the study of novel compounds. This knowledge is crucial in selecting the right drug candidate and the appropriate clinical dose.

## Materials and Methods

### Animals and administration of drug

C57BL/6 female mice (Harlan Laboratories, Horst, The Netherlands), Tg2576 transgenic female mice (Taconics, Georgetown, NY), overexpressing human APP with the Swedish mutation (Hsiao et al. 1996), and Tg2576 wild-type littermates were used in the experiments. The C57BL/6 and wild-type littermates were only used for pharmacokinetic PK sampling and analysis. The housing environment was humidity- and temperature-controlled with a constant 12-h light/dark cycle. The animals were kept in conventional housing (5–10 mice per cage) and had access to standard rodent chow and tap water ad libitum. The transgenic mice were aged at the local AstraZeneca (AZ) animal facilities. On the day of study, the mice were weighed. MRK-560 (synthesized in-house; AZ) or vehicle was administered by oral gavage. Three types of vehicles were used: (1) 1% MCC/NaCMC (Microcrystalline Cellulose/Sodiumcarboxymethyl Cellulose) +0.6% lipoid S100, (2) 20% HPβCD in 0.1 mol/L Meglumine, and (3) 40% HPβCD in 0.3 mol/L Meglumine. Observations of the animals’ health were made throughout all the experiments. Data from 23 MRK-560 studies were used in this analysis and details of all studies can be found in Table 1. For the AP analysis, data from vehicle and untreated Tg2576 mice from 48 additional (i.e., non-MRK-560) in-house studies were used. The additional dataset consisted of Aβ levels measured in Tg2576 mice of various ages, between 3.5 and 26 months at termination. Details of those data can be found in Appelkvist et al. (2011). In total, data from 71 preclinical studies were used.

**Table 1:** A summary of study designs for MRK-560 in-house studies

Study number	Species	Animal age at study start(weeks)	Number of animals (treatment + vehicle)	Treatment duration (days)	Sample collection (h since last dosing)	Doses (μmol/kg)	Route of administration	Vehicle
1	C57BL/6	10	9 + 9	7	4.5, 172.5	30	p.o.	1
2	C57BL/6	14	6 + 3	4	3	7.5, 30	s.c.	2
3	C57BL/6	15	12 + 6	4	3	7.5, 30	s.c.	2
4	C57BL/6	N.A. (PK study)	3	1	0.5, 1.5, 3, 5, 7, 24	10	s.c.	2
5	C57BL/6	N.A. (PK study)	6	1	0.016, 0.08, 0.36, 0.66, 1, 3, 6, 24, 48	3, 10	p.o. and i.v.	1
6	TG littermates	28	15 + 10	28	4.5	30	p.o.	1
7	Tg2576	20	10 + 10	4	3	70	p.o.	1
8	Tg2576	24	30 + 15	30	3	0.5, 6	p.o.	1
9	Tg2576	24	30 + 15	94	3	0.5, 6	p.o.	1
10	Tg2576	24	74 + 30	29	3	1, 3, 6	p.o.	1
11	Tg2576	24	16 + 16	30	3	6	p.o.	1
12	Tg2576	25	6 + 6	1	3	70	p.o.	1
13	Tg2576	25	24 + 6	4	3	1, 3, 7.5	p.o.	1
14	Tg2576	25	30 + 30	1	3, 6, 24, 48, 120	70	p.o.	1
15	Tg2576	36	20 + 20	8	3	6	p.o.	3
16	Tg2576	60	27 + 27	4	3	6	p.o.	1
17	Tg2576	68	11 + 10	4	3	6	p.o.	1
18	Tg2576	81–89	80 + 80	1	6, 24, 48, 96	70	p.o.	1
19	Tg2576 and TG littermates	32	12 + 12	4	4.5	30	p.o.	1
20	Tg2576 and TG littermates	32	12 + 8	21	4.5	30	p.o.	1
21	Tg2576 and TG littermates	36	30 + 30	8	3	6	p.o.	3
22	Tg2576 and TG littermates	24–28	34 + 12	45	4.5	30	p.o.	1
23	Tg2576 and TG littermates	37–40	16 + 16	28	4.5	30	p.o.	1

Vehicle corresponds to: (1) 1% MCC/NaCMC+0.6% lipoid S100, (2) 20% HPβCD in 0.1 mol/L Meglumine, and (3) 40% HPβCD in 0.3 mol/L Meglumine.

All work involving animals was conducted according to the guidelines set by the Stockholm Animal Research Ethical Committee.

### Blood sampling

Blood samples were collected by tail vein puncture in PK studies and occasionally in efficacy studies. At termination blood was withdrawn from anesthetized mice by heart puncture into prechilled microtainer Ethylenediaminetetraacetic acid tubes. Blood samples were immediately put on ice and centrifuged for 10 min at ∼3000*g* at +4°C within 20 min from sampling. Plasma was transferred to prechilled polypropylene tubes. The tubes were immediately frozen on dry ice and stored at −70°C until analysis.

### Brain dissection

After blood sampling, mice were sacrificed by decapitation followed by brain samples collection. Cerebellum and olfactory bulbs were removed and the forebrain was divided into left and right hemispheres, weighed, and snap frozen.

### Soluble and insoluble extraction of brain tissue

Left hemisphere of the cerebrum was sequentially extracted by diethylamine (DEA) followed by formic acid (FA) to obtain soluble and insoluble fractions of Aβ species, respectively. In short, brain tissue was sonicated in 1:18 (w/v) 0.2% DEA and 50 mmol/L NaCl, pH 11.6. After centrifugation (133,000*g*, 4°C, 1 h), the supernatant (soluble Aβ) was recovered, neutralized to a pH of 8.0 using 2 mol/L Tris-HCl, and immediately frozen to -70°C. The remaining pellet was sonicated in 1:18 (w/v) 70% FA. Homogenates were centrifuged at +4°C for 1 h at 133,000*g*. Recovered supernatants (insoluble Aβ) were neutralized to pH 7.5 with 1 mol/L Tris at 1:20 (w/v) dilutions, frozen on dry ice, and stored at −70°C until analysis.

### Aβ analysis

Amyloid beta (Aβ40) and Aβ42 concentrations were measured in plasma, in soluble (DEA), and insoluble (FA) brain fractions using commercial sandwich ELISA kits (KHB3482 from Invitrogen, Carlsbad, CA, and RUO80177 from Innogenetics, Gent, Belgium). To compensate for matrix effects, plasma or brain homogenates from non-transgenic mice were used for preparation of standard curves. The lower and higher limits of quantification (LLOQ and HLOQ) were determined for each immunoassay plate based on the lowest and highest standard point, respectively. The coefficient of variation was less than20% and the accuracy was between 80% and 120%.

### Bioanalysis

Plasma samples and standards were precipitated with acetonitrile containing internal standard. After centrifugation, supernatant was transferred to a new 96-well plate, diluted with mobile phase, and injected on the LC/MS/MS system (Waters Corporation, Sollentuna, Sweden) (Bueters et al. 2011). For the determination of the compound concentration in the brain, frozen mouse brains were weighed, and an ice-cold Ringer solution (two volumes per weight) was added. The brains were sonicated using a Multi-element probe SONICS VCX 500 (Newtown, CT). To 50 μL homogenized tissue 150 μL ice-cold acetonitrile containing an internal standard was added in a precipitation plate (96-well PP-plate; Waters, Milford, MA). After mixing and centrifugation (4°C, 4000 rpm, 20 min), supernatant was transferred to an analysis plate (PP-plate, Waters) and analyzed by LC/MS/MS (Borgegård et al. 2011). As the brains were not perfused before exposure analysis, a volume of 1.3% of blood in the brain and the concentration of compound in plasma were used to calculate the brain concentration (Richard 1968; Park and Sinko 2005).

Determination of the fraction of compound unbound in the brain was determined in a rat brain slice uptake method (Fridén et al. 2009; Borgegård et al. 2011). In short, male Sprague-Dawley rats were decapitated under isoflurane anesthesia. The brains were immersed in an ice-cold oxygenated extracellular fluid (ECF) buffer. Coronal slices of the striatal areas (300 μm) were preincubated in a 10 mL ECF buffer for 5 min at 37°C followed by incubation with 1 μmol/L compound in an ECF buffer for 5 h at 37°C under 5% CO_2_ in oxygen. After incubation, brain slices were weighed and homogenized in nine volumes (w/v) of ECF buffer. The slice homogenates and ECF buffer were stored at −20°C prior to analysis. To 50 μL homogenized tissue, 150 μL ice-cold acetonitrile containing an internal standard was added in a precipitation plate. After mixing and centrifugation (4°C, 4000 rpm, 20 min), supernatant was transferred to the analysis plate and analyzed by LC/MS/MS.

### Modeling of MRK-560 pharmacokinetics

In the pharmacokinetic analysis, one- and two-compartmental first-order models were evaluated to fit the MRK-560 concentration–time profiles in plasma and brain. Separate absorption rate constants were estimated for oral and subcutaneous route of administration. A dose-dependent bioavailability was assessed and was modeled using the function shown in equation (1):

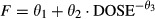
(1)

The *θ*_1_ parameter was estimated separately for different studies in the case where it resulted in the improvement of the model (based on the analysis of the objective function).

An instantaneous distribution of the unbound compound between plasma and brain was assumed. The brain exposure was modeled using equation (2):

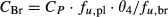
(2)where *C*_*P*_ was the plasma concentration, *θ*_4_ was the brain:plasma concentration ratio, and *f*_*u,*pl_ and *f*_*u,*br_ were the plasma and brain unbound fraction, respectively. The 2.5% of plasma and 4.5% of brain concentration measurements were below limit of quantification and were excluded from the analysis. Predicted individual total brain concentrations were used as an input for the population PD model.

### Modeling of age-dependent AP

In a first step, the influence of age on Aβ levels was assessed using data from naïve and vehicle-treated animals only. The descriptive analysis was performed using a logistic model (Van der Graaf and Schoemaker 1999) as shown in equation (3):

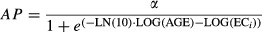
(3)where *α* was the upper asymptote, EC_*i*_ was a point of inflection and *P* was the slope parameter.

### Integrated model of age-dependent AP and age-dependent drug effect

In a second step, drug effect data were added to the AP analysis in order to develop an integrated PKPD model that incorporated both age-dependent AP and an age-dependent drug effect. To this end, the initially developed logistic model (eq. 3) was combined with a turnover model (Dayneka et al. 1993; Jusko and Ko 1994). This part of the analysis was only performed for soluble and insoluble Aβ40, as drug effect data for Aβ42 were not available from all studies. In this approach, the age-dependent AP causing an increase in soluble Aβ40 production (*K*_*in,*sol_) was described following equations (4 and 5):

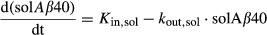
(4)

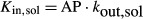
(5)in which *K*_in,sol_ and *k*_out,sol_ corresponded to the production and loss of soluble Aβ40, respectively.

The inhibitory effect of MRK-560 on soluble Aβ40 was modeled using a turnover model (eq. 4), where the production of soluble Aβ40 was inhibited by the drug concentration. The model took into account the time delay between changes in plasma concentration of the drug and changes insoluble Aβ40 levels. Three types of drug effect models were evaluated: (A) absolute (eq. 6), (B) relative change from the baseline (eq. 7), and (C) modified relative change from the baseline (eq. 7). In model B the IC_50_(sol*Aβ*40) was constant, while in model C it was dependent on the soluble Aβ40 levels via a linear function as shown in equation (8).



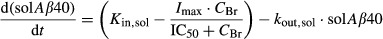
(6)

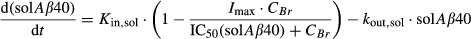
(7)

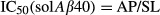
(8)

*K*_in,sol_ corresponded to the age-dependent production of soluble Aβ40, *k*_out,sol_ corresponded to the loss of soluble Aβ40, *I*_max_ was a maximum drug-induced soluble Aβ40 reduction (either absolute or relative reduction from the baseline for eqs. 6 and 7, respectively), *C*_Br_ represented the brain concentrations, IC_50_ was a concentration which induced half of the maximum inhibition, while *AP* corresponded to the AP, described using equation (3). Additionally, parameter SL represented a slope of the linear relationship between absolute Aβ40 levels and IC_50._

**Figure 1:**
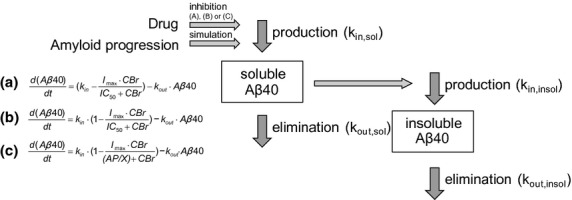
A schematic representation of the proposed amyloid progression (AP) and MRK-560 drug effect model. The AP is simulating production of soluble Aβ40(*k*_in,sol_), while the drug is causing its inhibition. Three models for drug inhibition were investigated – A, B, or C, of which C was found to best describe in-house and literature data (see Discussion). The levels of soluble Aβ40 are in turn affecting the production of insoluble Aβ40 aggregates (*k*_in,insol_).

Changes in soluble Aβ40 were assumed to change the production of insoluble Aβ40. This effect was modeled using a turnover model and was described using equations (9–11):

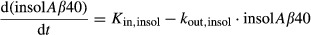
(9)

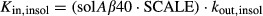
(10)

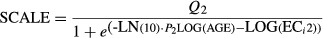
(11)where *K*_in,insol_ was the production of insoluble Aβ40, which was dependent on the soluble Aβ40 levels, *k*_out,insol_ was the loss of insoluble Aβ40, and SCALE was the scaling function between soluble and insoluble Aβ40 levels. Similar as in equation (3), *α*_2_ from equation (11) was the upper asymptote in the logistic function, EC_*i*2_ was a point of inflection and *P*_2_ was the slope parameter. A graphical representation of the PKPD model can be found in Figure 1.

### Modeling procedures

All modeling analyses were performed in NONMEM (version 7.1.0, ICON Development Solutions, Ellicott City, MD [Schoemaker and Cohen 1996]). The best models were chosen based on the analysis of their objective function, the precision of parameter estimates, and goodness-of-fit plots. The analysis of NONMEM output was performed using R software (R Development Core Team 2008) and Xpose4 package (Jonsson and Karlsson 1998). The 90% prediction intervals were calculated using the visual predictive check (VPC) as described by (Post et al. 2008). In brief, 1000 simulations of the data were performed using final parameter estimates and 5th, 50th, and 95th percentiles were calculated.

### Statistical analysis

Statistical comparisons of the vehicle and MRK-560 treatment data were made by unpaired *t*-tests. The level of significance was set at *P* < 0.05. All analyses were performed using GraphPad Prism (GraphPad Prism Software Inc., San Diego, CA) and results were reported as mean ± SEM.

## Results

### Modeling of MRK-560 pharmacokinetics

**Figure 2:**
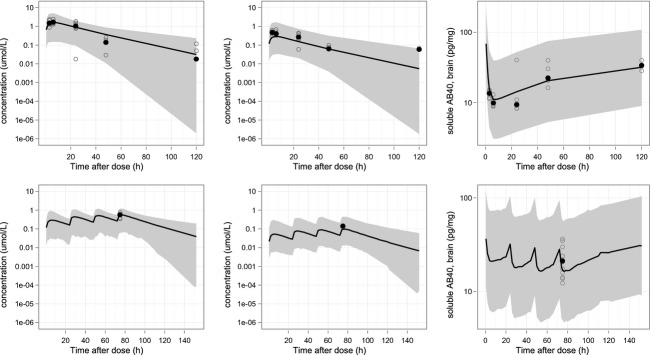
Examples of pharmacokinetic and pharmacodynamic profiles following oral administration of a single dose (study #14, 70 μmol/kg – top panel) and a 4-day treatment (study #13, 7.5 μmol/kg daily dose – bottom panel) of MRK-560 to the Tg2576 mice. Left and middle panels show profiles of plasma and brain exposures, respectively. The right panel represents measured soluble Aβ40 levels and a pharmacodynamic profile predicted using model C. The plots show the actual measurements (gray open circles), median (black circles), and model predicted profiles (black lines) with 90% prediction intervals (gray area).

Plasma and brain exposure data from C57BL/6, Tg2576, and Tg2576 wild-type littermates were used in the PK analysis. The observed *f*_*u,*pl_ and *f*_*u,*br_ values were 0.4% and 0.67%, respectively. The estimated total brain:plasma ratio of 0.3 was consistent with the observed brain:plasma ratio and constant over a concentration range between 0.01 and 15 μmol/L. A two-compartmental model with first-order absorption and elimination was found to best describe the concentration–time profile in plasma. A fourfold higher plasma exposure was observed with vehicles 2 and 3 (20% HPβCD in 0.1 mol/L Meglumine and 40% HPβCD in 0.3 mol/L Meglumine), compared to vehicle 1 (1% MCC/NaCMC+0.6% lipoid S100). Therefore, the bioavailability was estimated separately for studies in which vehicles 2 and 3 were used. For vehicle 1, the bioavailability was found to be dose-dependent and was modeled using equation (1). The parameter estimates of *θ*_1_ ranged from 0.0001 to 0.2. No differences in MRK-560 pharmacokinetics were found among C57BL/6, Tg2576, and Tg2576 wild-type littermates. All final parameter and variability estimates are found in Table 2. Model-predicted pharmacokinetic profiles, plotted on top of the experimental data from two representative studies (a single dose and a 4-day dosing study) are shown in Figure 2. Additionally, goodness-of-fit plots are presented in the Supplementary Material (Fig. S1).

**Table 2:** Summary of pharmacokinetic parameter estimates for MRK-560

Parameter	Units	Parameter estimate	BSV (%)
*K*_A_ (oral)	1/h	0.41	–
*K*_A_ (s.c.)	1/h	0.81	–
CL	l/h/kg	0.12	–
*V*_2_	l/kg	0.28	179
*Q*	l/h/kg	7.6	–
*V*_3_	l/kg	2.9	50
Brain:plasma ratio	–	0.31	61
Dose-dependancy function of bioavailability (vehicle 1)			
θ_1_*	–	0.0001-0.2	–
θ_2_	–	0.22	–
θ_3_	–	0.5	–
Bioavailability for p.o. formulation with vehicle 2, 3, and sc formulation			
F_veh2_	–	1	–
F_veh3_		0.7	–
F_sc_	–	0.8	–

In case of values indicated with *, given parameters were estimated separately for different studies. Vehicle 1, 2, and 3 corresponds to 1% MCC/NaCMC+0.6% lipoid S100, 20% and 40% HPβCD in 0.1 mol/L Meglumine, respectively (for details, see Materials and Methods).

### Modeling of AP

**Figure 3:**
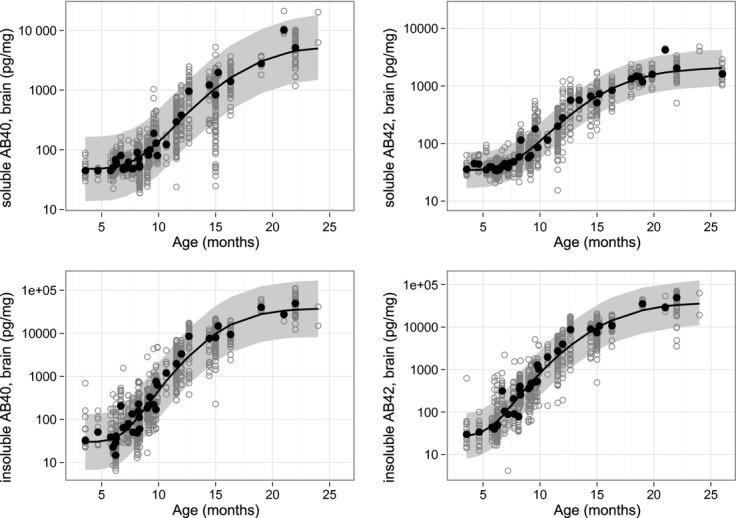
Exponential increase in soluble Aβ40 and 42 (top panels) and insoluble Aβ40 and 42 (bottom panels). The plots show the actual measurements from vehicle and untreated animals (gray open circles), median (black circles), and model predicted profiles (black lines) with 90% prediction intervals (gray area). The *x*-axis corresponds to the age at termination (months).

Amyloid progression was initially assessed using data from naïve and vehicle-treated animals with an age range of 3.5–26 months. The analysis was performed for both soluble and insoluble Aβ40 and Aβ42 variants. A continuous increase in Aβ levels with age was observed for the four Aβ variants, as shown in Figure 3. After an initial constant phase in young animals (<7 months), the absolute Aβ levels increased exponentially over time and reached a plateau in old animals (>20 months). The age-dependent increase in Aβ levels was best described using a logistic model (eq. 3). Using this function, it was possible to describe the initial baseline values, plateau levels (the upper asymptote parameter *α*), and the shape of the exponential increase (the slope parameter *P*). The initial baseline values and the plateau levels were observed to be different for the Aβ variants. Soluble Aβ40 levels rose from 47 pg/mg in young to 6640 pg/mg in old animals (plateau of Aβ accumulation), while insoluble Aβ40 levels changed from 30 to 39,900 pg/mg (Fig. 3). This corresponded to a 141- and 1330-fold increase over the age range for soluble and insoluble Aβ, respectively. The insoluble Aβ40 increased ∼10-times more than soluble Aβ40 during the AP. The point of inflection (EC_*i*_) corresponded to 50% of the maximum Aβ concentration and was reached between 17 and 20 months (Table 2). The final model predictions, prediction intervals, and observations are shown in Figure 3. The final parameter estimates for each Aβ variants are presented in Table 3.

**Table 3:** Summary of age-dependent amyloid progression parameter estimates for soluble, insoluble Aβ40 and Aβ 42

		Parameter estimate			
		
Parameter	Units	Soluble Aβ40	Insoluble Aβ40	Soluble Aβ42	Insoluble Aβ42
*α*	pg/mg	6640	39,900	2240	40,900
EC_*i*_	Months	20	17	18	18
*P*	–	6.4	8.3	5.9	6.8
Baseline	pg/mg	48	29	34	27

Parameters were estimated usingTg2576 animal data after vehicle administration and from untreated animals only.

### Modeling of age-dependent GSI effect on soluble and insoluble Aβ40 levels

**Figure 4:**
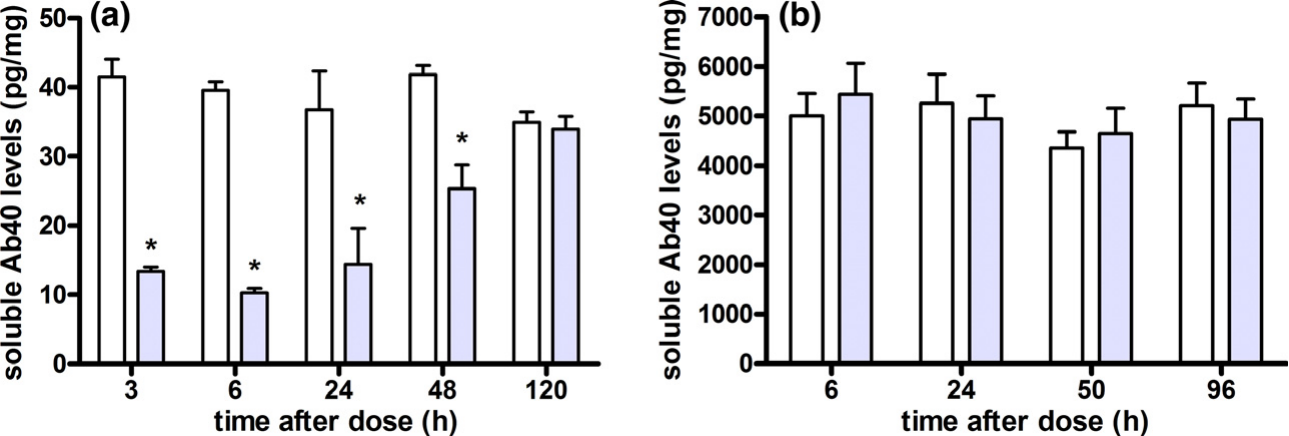
Soluble brain Aβ40 time courses in (A) young (study #14, 5.5-month-old) and (B) old (study #18, 18 months old) Tg2576 mice treated with a single oral dose of 70 μmol/kg MRK-560. White boxes correspond to the average (+SEM) vehicle levels, while light gray boxes represent dose treatment. *indicates that the effect was statistically significant (determined using unpaired *t*-test).

A detailed summary of the MRK-560 effects on soluble and insoluble Aβ40 concentrations from each study is presented in Table 4. In Figure 2, relevant examples on the inhibitory effects of MRK-560 are shown for a single dose and for a 4-day treatment. It was observed that MRK-560 caused concentration-dependent inhibition of soluble Aβ40 levels in animals up to 9 months old, but not in animals over 13 months old (Fig. 4). It was observed that a 70 μmol/kg single dose of MRK-560 caused a reduction of 29 pg/mg (74% decrease from baseline) in soluble Aβ40 levels in young mice 6 h after dosing. In old animals the same dose of MRK-560 did not reduce Aβ40 levels at any time-point after dosing. The baseline Aβ levels were more than 100-fold higher in the latter experiment. For mice with age between 9 and 13 months, no data were available (see Table 4).

**Table 4:** A summary of MRK-560 effects on soluble and insoluble Aβ40 levels

Study number	Doses (μmol/kg)	Soluble Aβ40, vehicle (pg/mg), mean ± SEM	Soluble Aβ40, treatment (pg/mg), mean ± SEM	Effect on soluble Aβ40 (%)	Insoluble Aβ40, vehicle (pg/mg), mean ± SEM	Insoluble Aβ40, treatment (pg/mg), mean ± SEM	Effect on insoluble Aβ40 (%)
7	70	82 ± 3	23 ± 1	−72*	–	–	–
8	0.5	49 ± 2	46 ± 2	−6	48 ± 5	45 ± 5	−8
	6	49 ± 2	26 ± 1	−45.6*	48 ± 5	21 ± 3	−57*
9	0.5	65 ± 9	54 ± 3	−17	147 ± 45	152 ± 21	3
	6	65 ± 9	46 ± 6	−28	147 ± 45	194 ± 58	32
10	1	64 ± 2	47 ± 1	−26*	16 ± 1	9 ± 1	−40*
	3	64 ± 2	38 ± 1	−41*	16 ± 1	9 ± 2	−42*
	6	64 ± 2	34 ± 2	−47*	16 ± 1	5 ± 1	−69*
11	6	62 ± 3	37 ± 2	−40*	33 ± 3	23 ± 2	−31*
12	70	40 ± 2	10 ± 2	−76*	–	–	–
13	1	47 ± 2	28 ± 3	−41*	–	–	–
	3	47 ± 2	21 ± 1	−56*	–	–	–
	7.5	47 ± 2	14 ± 1	−69*	–	–	–
14	70	40 ± 1	10 ± 1	−74*	–	–	–
15	6	64 ± 4	32 ± 7	−50*	214 ± 25	218 ± 44	2
16	6	1771 ± 143	1461 ± 122	−17	–	–	–
17	6	1989 ± 96	1863 ± 197	−6.3	14,400 ± 640	13,310 ± 874	−7.6
18	70	5003 ± 447	5432 ± 630	8	–	–	–
19	30	49 ± 2	22 ± 1	−55*	–	–	–
20	30	57 ± 10	17 ± 1	−70*	96 ± 34	29 ± 4	−70
21	6	80 ± 6	30 ± 11	−63*	333 ± 57	233 ± 78	−30
22	30	59 ± 12	25 ± 4	−57*	68 ± 9	29 ± 3	−57*
23	30	101 ± 9	45 ± 8	−56*	220 ± 54	72 ± 13	−67*

A dash indicates that no measurements were performed.

* The effect was statistically significant (*P* < 0.05)

**Figure 5:**
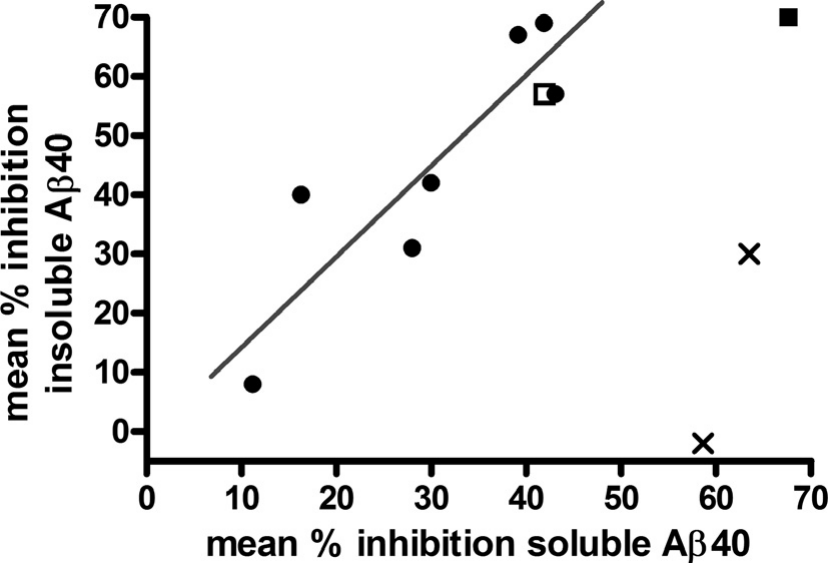
Relationship between the model-predicted reduction in solubleAβ40 at steady state and the observed reduction in insoluble Aβ40 in young animals. The soluble Aβ profile was simulated over the duration of the treatment at the specific age of the mice. The average% reduction in soluble Aβ40 from the predicted amyloid progression at time of termination was derived and plotted against the observed percent reduction in insoluble Aβ40 as listed in Table 1 (Study 8, 10, 11, 15, 20, 21, 22, 23). Treatment duration is indicated by symbol: 1 week (crosses), 3 weeks (closed squares), 4 weeks (closed circles), and 6 weeks (open squares) of MRK-560 treatment. The regression line was fitted to the data points from 4–6 week treatment.

Insoluble Aβ40 levels were reduced after repeated dosing of MRK-560. At least 3 weeks of treatment were needed to demonstrate significant reductions in insoluble Aβ40. The model-predicted average reduction in soluble Aβ40 was calculated for the entire treatment period and plotted against the observed reduction in insoluble Aβ40 in Figure 5. Due to the short turnover rate of soluble Aβ40, soluble Aβ40 changed over the day following the plasma concentration with a short delay. For that reason, the observation for soluble Aβ40 at the time of termination (3 or 4 h after last dose) was not necessarily reflecting the average reduction during the whole treatment period. For that reason, the model-predicted average reduction was calculated for the full treatment period for each study. Insoluble Aβ40 levels were not changing rapidly based on drug plasma concentrations due to the slow turnover rate. Therefore, the observed insoluble Aβ40 levels were used in the plot. Figure 5 shows the linear relationship between reduction in soluble and insoluble Aβ40 was dependent on duration of treatment. A different slope was found for the results from 4 to 6 weeks of treatment compared to 1–3 weeks. This indicated that treatment duration of 1 month is needed to maximize the effects on insoluble Aβ40. The 12-week treatment group was not plotted in Figure 5 as the observed effects for both soluble and insoluble were not significant.

The modeling of the changes in soluble and insoluble Aβ40 levels following drug administration took into account the influence of age-dependent AP and the drug effects. The AP was assumed to stimulate soluble Aβ40 production, while the drug was assumed to cause its inhibition. The age-dependent AP was re-fitted using all available data with drug effect measurements. The drug effect was assessed using three types of models: A, B and C (see Figure 1). The final parameter estimates for model C can be found in Table 5. Final parameter estimates for models A and B are presented in Supplementary Material (Table S1). Representative examples on model fits by model C are shown in Figure 2. The goodness-of-fit plots are shown in Supplementary Material (Fig. S1). An additive residual error model was estimated separately for soluble and insoluble Aβ40 measurements. Using the final model, he turnover rate of insoluble Aβ40 (*k*_out,insol_) was estimated at 0.001 1/h, corresponding to a turnover half-life of ∼30 days. The turnover rate of soluble Aβ40 (*k*_out,sol_) could not be accurately estimated. Therefore, this parameter was fixed at the value of 1.1 h^−1^ (i.e., a half-life of 38 min) as reported by a literature review of the Aβ40 turnover in the brain (Barten et al. 2005). In the final model, the *I*_max_ value was estimated at 86%, and the slope of the linear relationship between Aβ40 levels and IC_50_ was estimated at 514. According to this relationship, the IC_50_ was 0.11 μmol/L in 5-month-old mice and gradually increasing to 12 μmol/L in 25--old mice.

**Table 5:** Summary of pharmacodynamic parameter estimates for model C

Parameter	Units	Parameter estimate ± SE
Soluble Aβ disease progression		
Alpha	pg/mg	6620 ± 472
EC_*i*_	Months	18.1
*P*	–	7.5 ± 0.2
Drug effect		
Baseline, soluble	pg/mg	57.1 ± 0.9
*K*out,soluble	1/h	1.1
Half-life Aβ,soluble	Min	38
*I*_max_	pg/mg	0.86 ± 0.04
IC_50_ slope (*X*)	–	514 ± 99
Insoluble Aβ		
*K*out,insoluble	1/h	0.001 ± 0.0002
Half-life Aβ,insoluble	Days	30.5
Baseline,insoluble	pg/mg	28.9
Scaling between soluble and insoluble Aβ		
Alpha2	pg/mg	6.5 ± 0.2
EC_*i*2_	h	7610 ± 87
*P*_2_	–	9.1 ± 0.6
Residual error		
Soluble Aβ	%	51
Insoluble Aβ	%	83

Parameters were estimated using all available Tg2576 animal data, including data from untreated animals as well as after vehicle and drug administration.

**Figure 6:**
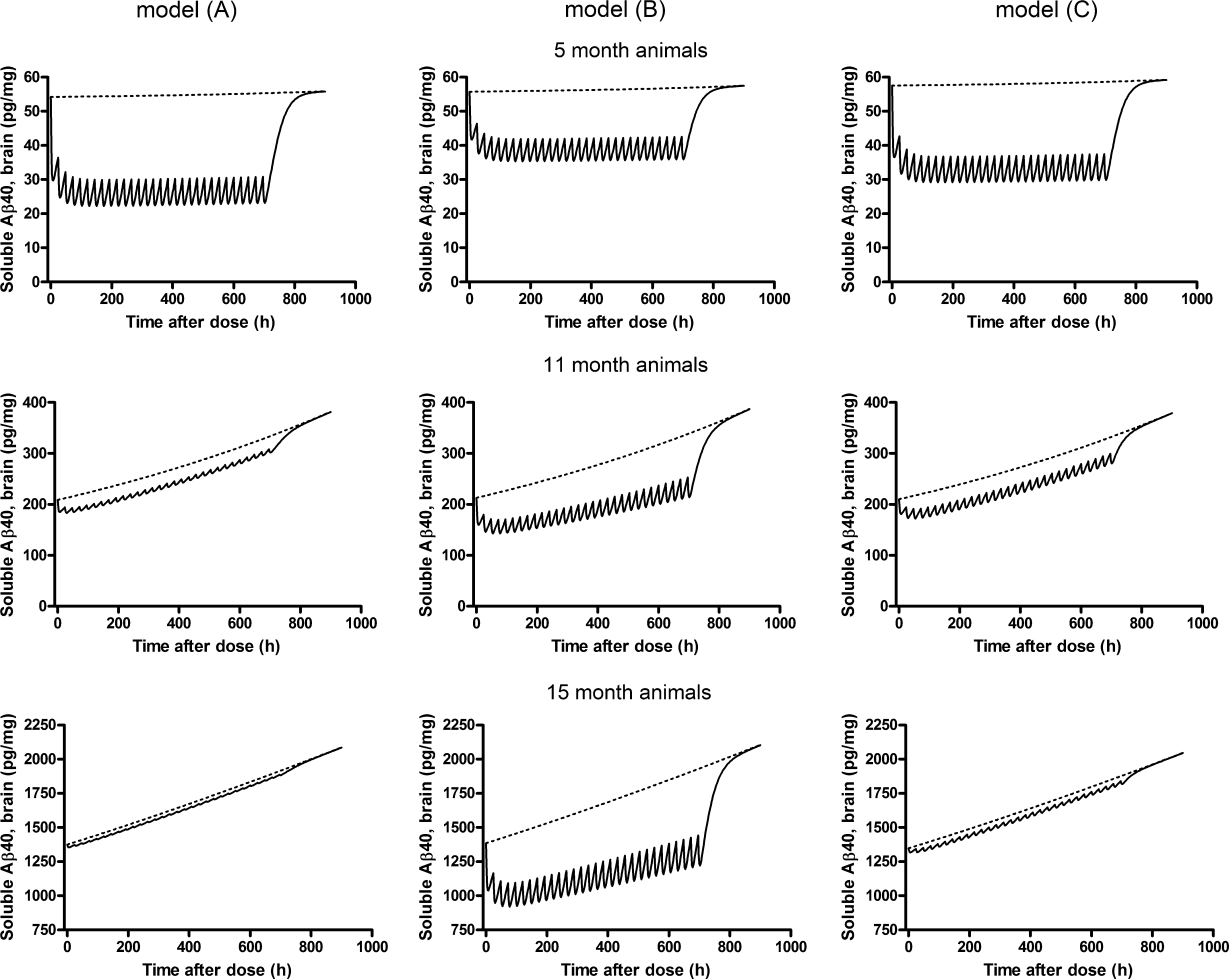
Simulations of 1-month MRK-560 treatment with daily dose of 6 μmol/kg in young (5-month old), middle (11-month old) and old (15-month old) Tg2576 mice for models A, B, and C. Details of each model can be found in the Materials and Method section. Dotted lines represent amyloid progression, while drug effect is shown as a solid line.

**Figure 7:**
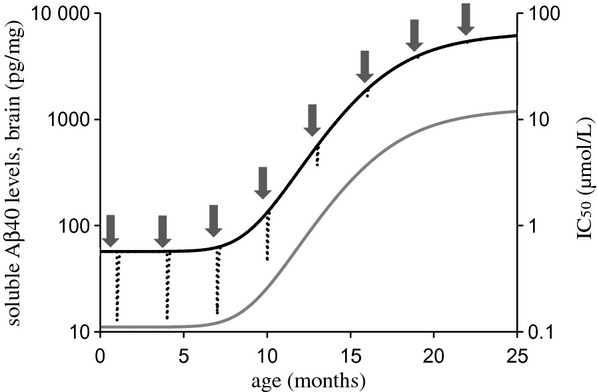
A simulation of an effect of a single 70 μmol/kg dose of MRK-560 administered to animals every 3 months. The graph illustrates the amyloid progression (solid line), effect of MRK-560 (dotted line) as well as the Aβ-dependent change in IC_50_ (gray, solid line, right *y*-axis). The simulation was performed using final parameter estimates from model C described in the text. The arrows indicate the times of single-dose administration.

To discriminate between Aβ40 profiles predicted by model A, B, and C, simulations of 1-month MRK-560 treatment were performed for young (5-month old), middle (11-month old) and old (15-old) animals (Fig. 6). The model predictions were similar in young animals, whereas simulations in middle and old animals allowed model discrimination. Based on these results, model C was chosen as the one that best described all data. Additional simulations of single-dose effects in mice of different ages were performed using final parameter estimates from model C and are shown in Figure 7.

## Discussion

The first aim of the study presented here was to quantify the age-dependent Aβ accumulation in the transgenic Tg2576 mouse model. To this end, a semi-mechanistic PKPD modeling approach was used to successfully describe the exponential AP for four Aβ variants (soluble and insoluble brain Aβ40 and Aβ42) in mice with increasing age. The second aim was to quantify the influence of age-dependent AP on the efficacy of the γ-secretase inhibitor, MRK-560, in mice of different ages. A model was developed that was able to describe the age- and treatment-dependent effects of MRK-560 on soluble and insoluble Aβ40 levels, while taking into an account the continuous rise in Aβ levels with age. The third aim was to investigate the influence of treatment duration on the lowering of insoluble Aβ. The estimated insoluble Aβ40 turnover half-life of 30 days demonstrated that at least 1 month of treatment is needed to lower insoluble Aβ substantially.

Modeling of disease progression has been rather limited to date, partly because the natural course of most diseases is often poorly understood (for a review see: Schmidt et al. 2011). In general, disease progression can be regarded as a disruption of the natural balance in a biological system. Neglecting the underlying disease progression may lead to an inaccurate characterization of the treatment effect, resulting in an incorrect dose selection and poor study design. Mathematically, disease progression can be expressed by a turnover model (Dayneka et al. 1993) where either production or elimination of a given biomarker is altered during the disease course.

The development of AD is characterized by a continuous progression of the disease. The Tg2576 mouse model used displays Aβ accumulation as one of multiple factors in the progression of AD pathology.To our knowledge, the AP has not been quantitatively described to date. Our AP models described the continuous progress for four Aβ variants. The shape of the fitted function was sigmoidal, with initial lag phase, where no increase in Aβ levels was observed, followed by an exponential increase and reaching a plateau phase at a later age (see Fig. 3). These observations were in agreement with other reports (Kawarabayashi et al. 2001; Das et al. 2012; Rogers et al. 2012).

In the final model, age changed the production of Aβ as is shown schematically in Figure 1. Tg2576 mice overexpress a Swedish mutation variant of human APP (Hsiao et al. 1996), resulting in an altered production of Aβ. In comparison to normal cells, the Aβ production was increased 6–8 times in the cultured cells that express mutated β-APP (Citron et al. 1992). This increased production was linked to the altered cellular mechanism (Haass et al. 1995). It appears biologically plausible that AP is acting on synthesis rate, rather than elimination rate of Aβ. It is possible that an increased Aβ production is not the sole factor underlying AP in the Tg2576 mouse. Changes in Aβ elimination could potentially also contribute. For example, recent findings showed that levels of sAPPβ_SWE_ and sAPPα were constant over time in Tg2576 mice, which implies that Aβ accumulation may be due to the changes in its clearance rather than production (Eketjäll et al. 2013). Therefore, in the model development, the scenario was explored where AP affected Aβ elimination. Such model could not describe the relationship between soluble and insoluble Aβ40 levels (data not shown), suggesting that an altered production rate of Aβ over time was most plausible.

In the quantification of the age-dependent efficacy of MRK-560, three models were evaluated. Model A used an absolute drug effect, model B used a relative drug effect, and model C used a relative effect with Aβ-dependent IC_50_ to describe the concentration–effect relationship. Model A could accurately describe the MRK-560 effect in both young and old animals. In young animals the absolute effect represents almost 100% inhibition. In contrast, a similar effect size is only 5% of the baseline value in old mice and falls within the measurement error. Model A could also describe some of the literature findings. For example, Barten et al. (2005) reported a large decrease in Aβ40 brain levels in 3–6 month animals, and no effect in animals between 14–17 months old. Other authors reported large decreases in Aβ levels in young Tg2576 mice after γ-secretase modulator treatment (Van Broeck et al. 2011; Rogers et al. 2012). However, there are also reports that are not consistent with model A. In 12-month-old Tg2576 mice, Townsend et al. (2010) reported a 63% decrease in soluble Aβ40 levels after a 3-month MRK-560 treatment and Best et al. (2007) showed a 43% decrease in total Aβ40 levels. Model A would not predict an effect in these animals due to their high Aβ40 baseline levels.

Model B could only describe observations seen in young animals. The model predicts large drug effects regardless of Aβ40 baseline levels (Fig. 6). This is not consistent with in-house data from old animals where no reductions in Aβ40 were observed after MRK-560 treatment. Model C described the MRK-560 effect in both young and old animals, and in literature. According to this model, IC_50_ was found to increase from 0.11 μmol/L in 5-month to 12 μmol/L in 25-month Tg2576 mice. In other words, the model implies a gradual decrease in drug efficacy during AP (Fig. 7). The same single dose of the drug has a different effect on Aβ40 depending on animal age. Consequently, the model could describe the drug effect in young and old animals. In young animals, the Aβ levels were low and the drug produces large Aβ reductions. In old animals the Aβ levels were high, drug efficacy was decreased, and the same concentration of the drug produced Aβ reduction that fell within the measurement error. Model C also explains results from literature studies in which Aβ reductions were observed in old animals (Best et al. 2007; Townsend et al. 2010). As the drug efficacy depends on the absolute Aβ levels, it is possible that the Tg2576 mice used in these published studies had different Aβ baseline levels compared to the animals used in this study. Absolute Aβ levels are dependent on different brain extraction methods and/or assays, and cannot directly be compared between laboratories. It can be hypothesized that if the Aβ levels in these studies were lower, drug efficacy would not be greatly decreased, therefore, the drug would still cause large reductions in Aβ levels. Model C with the Aβ-dependent IC_50_ change could also explain a recent finding published by Das et al. (2012), in which a GSI displayed a progressively decreasing efficacy in Tg2576 mice. A 3-month GSI treatment was given to 4-, 7- and 12-month-old mice. Reduction in insoluble Aβ levels and plaque burden was greatest in 4-month, lower in 7-month, and not significant in 12-month-old animals.

The semi-mechanistic approach applied allowed the estimation of the turnover rate of insoluble Aβ40 (*k*_out,insol_). The half-life of insoluble Aβ40 was ∼1 month. Such long half-life can explain that insoluble Aβ40 levels were significantly reduced following long-term treatment, but not following acute treatment (Fig. 5, Wang et al. 2012).

The findings presented here have important implications for future study design using Tg2576 mice. Specifically, the fact that the GSI efficacy is decreasing during AP imply that in order to achieve large Aβ reductions, the GSI treatment needs to be administered early, that is before Aβ levels start to accumulate.

## Abbreviations

AD: Alzheimer's disease
AP: Amyloid progression
APP: Amyloid precursor protein
Aβ: Amyloid beta
DEA: diethylamine
ECF: extracellular fluid
FA: formic acid
GSI: γ-secretase inhibitor
HLOQ: higher limits of quantification
LLOQ: lower limits of quantification
PKPD: pharmacokinetic-pharmacodynamic
VPC: visual predictive check

## Supporting Information

**Figure S1.** Goodness-of-fit plots for the final pharmacokinetic (top panel) and pharmacodynamic (Model [C], bottom panel) models. **Table S1.** Summary of pharmacodynamic parameter estimates for models (A) and (B). Parameters were estimated using all available Tg2576 animal data, including data from untreated animals as well as after vehicle and drug administration.
