# Supplementary material for: Modeling of age-dependent amyloid accumulation and γ-secretase inhibition of soluble and insoluble Aβ in a transgenic mouse model of amyloid deposition
Source: Pharmacol Res Perspect. 2013 Dec 5;1(2):e00012. doi: 10.1002/prp2.12 (PMC4186430; doi:10.1002/prp2.12)
Supplement: Supplementary file 1 — Figure S1. Goodness-of-fit plots for the final pharmacokinetic (top panel) and pharmacodynamic (Model [C], bottom panel) models. Table S1. Summary of pharmacodynamic parameter estimates for models (A) and (B). Parameters were estimated using all available Tg2576 animal data, including data from untreated animals as well as after vehicle and drug administration. [file prp20001-e00012-SD1.docx]

Table S1. Summary of pharmacodynamic parameter estimates for models (A) and (B). Parameters were estimated using all available Tg2576 animal data, including data from untreated animals as well as after vehicle and drug administration.

| Parameter | Units | Parameter estimate (SE%) | |
| --- | --- | --- | --- |
|  |  | Model (A) | Model (B) |
| *soluble amyloid progression* |  |  |  |
| α | pg/mg | 6540 (7) | 6820 (7) |
| ECi | months | 18 (2) | 18 (2) |
| P | - | 7.6 (3) | 7.5 (3) |
| Baseline,soluble | pg/mg | 54 (2) | 55 (2) |
| kout,soluble | 1/h | 1.1 | 1.1 |
| half life Aβ,soluble | min | 38 | 38 |
| *drug effect* |  |  |  |
| Imax | pg/mg | 59 (4) | - |
|  | % | - | 88 (10) |
| IC50 | µmol/L | 0.09 (13) | 0.19 (22) |
| *insoluble amyloid progression* |  |  |  |
| kout,insoluble | 1/h | 0.0008 (20) | 0.0012 (18) |
| half life Aβ,insoluble | days | 38 | 23 |
| Baseline,insoluble | pg/mg | 27 (8) | 30 (8) |
| α2 | pg/mg | 6.5 (4) | 6.3 (4) |
| ECi2 | h | 7530 ( 1) | 7600 (1) |
| P2 | - | 9.6 (6) | 8.9 (7) |
| *residual error* |  |  |  |
| soluble Aβ | % | 52 | 53 |
| insoluble Aβ | % | 85 | 82 |

Figure S1. Goodness-of-fit plots for the final pharmacokinetic (top panel) and pharmacodynamic (Model (C), bottom panel) models.
